# Nutrition therapy for adverse reactions to histamine in food and beverages 

**DOI:** 10.5414/ALX386

**Published:** 2018-09-01

**Authors:** I. Reese

**Affiliations:** Ernährungsberatung und -therapie, Munich

**Keywords:** adverse reactions to food, histamine, histamine-intolerance, diagnosis, diamine oxidase, nutrition therapy, quality of life

## Abstract

Adverse reactions to food are suspected in one third of the German population, but only 10% of these assumed hypersensitivity reactions can be clinically confirmed. While diagnosis of food allergies is fairly easy due to objective laboratory parameters, non-allergic hypersensitivity reactions are difficult to diagnose because these objective markers are lacking so far. Adverse reactions to histamine are often suspected to be the cause of a wide range of symptoms, especially when no allergic pathomechanism can be identified. In order to confirm such a suspicion, it is inevitable to validate a reproducible association between consumption of histamine-rich food and beverages and symptoms to identify causative agents and to exclude other disorders. Thereafter, avoidance tests should be performed on the basis of individual requirements. General advice with a lot of restraints is often unnecessarily strict. Nutrition therapy aims at a reduction of symptoms to a minimum while maintaining a high quality of life.

**German version published in Allergologie, Vol. 34, No. 3/2011, p. 152-158**

## Introduction 

Adverse reaction to histamine, frequently also termed “histamine intolerance”, is a widespread clinical picture, but diagnosis is difficult. To give consideration to all its varieties and diverse influencing factors the term “adverse reactions to histamine” will be used in this article. The assumption that adverse reactions to histamine might primarily occur due to an enzyme deficiency, like lactose intolerance or hereditary fructose intolerance, is contradicted by the fact that reported adverse reactions to ingested histamine are not always reproducible. The authors of a recently published double-blind, placebo-controlled multicenter study conclude from the results of provocation tests in patients with suspected adverse reactions to histamine “that the term histamine intolerance presumably represents a symptom complex that only in individual cases can be traced back to histamine alone” [14]. 

## Functions of histamine 

**Figure 1.**
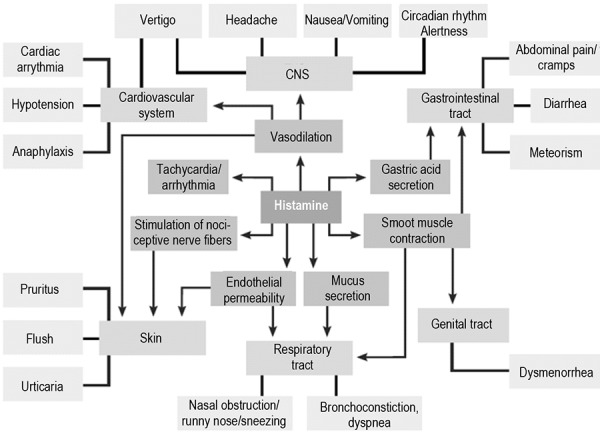
Histamine-mediated symptoms (according to Maintz et al. [16]).

Histamine is a transmitter with manifold important functions in the human body. As some effects of histamine can lead to medical conditions and thus are perceived as negative, it is often forgotten that histamine performs a number of physiological functions in the body. Diagrams on the mode of action of histamine frequently show symptoms (Figure 1), but do not impart its physiologically important effects. Its effects on the secretion of gastric acid, for example, are presented mainly by naming symptoms like abdominal pain, cramps, diarrhea or meteorism, while its positive effects in the sense of protein digestion are not mentioned. 

The finding that the intake of drugs inhibiting the secretion of gastric acid (proton pump inhibitors) can result in an incomplete protein digestion and thus in a reduced inactivation of allergens followed by an increased sensitization to allergens unstable to digestion [19, 28], illustrates the importance of taking into account all the effects of histamine. 

Its effects on female hormones are also frequently described as pathological, while they are in fact physiologically important. In this context Maintz et al. [17] could show that during pregnancy there is an upregulation not only of the histamine-depleting enzyme diamine oxidase, but also of the histamine-producing enzyme histidine decarboxylase. The authors conclude that the balance between histamine and diamine oxidase (DAO) is crucial for a complication-free pregnancy. 

Experiments in mice suggest that histamine plays an important role in the regulation of the energy balance too [7]. Mice that cannot synthesize histamine are characterized by visceral obesity, disturbed glucose tolerance, hyperinsulinemia and hyperleptinemia. 

In the context of immune responses histamine is not only a mediator of acute allergic and non-allergic reactions, but also acts as immunomodulator and influences chronic inflammation [2, 9]. 

## Histamine depletion 

Adverse reactions to histamine are explained by an insufficient histamine depletion. Histamine is depleted extracellularly by DAO, which is stored in vesicles and released if necessary, or intracellularly by histamine N-methyltransferase (HNMT). It is suggested that exogenous histamine is mainly depleted by DAO, although some authors also consider the depletion by HNMT to be of importance [15]. The latter assumption is, however, contradicted by results from an experiment in pigs to which the description of adverse events to histamine is traced back [24]: in this experiment the administration of 60 mg of histamine resulted in severe symptoms or death in three pigs with specifically inhibited DAO. Pigs with normal DAO activity tolerated cheese without any problems. A second experiment showed that premedication with a combination of H1- and H2-receptor antagonists could prevent symptoms [25]. 

These experiments were the first basis for the assumption that an insufficient DAO activity was the most important factor for the negative effects of histamine. While in the beginning the focus was put on the inhibition of DAO activity by drugs [23, 25], an underlying enzyme deficiency, detectable by blood tests, was assumed later [18]. Today it is well known that the parameters of DAO activity in the blood do not correlate with those in the intestine [8] so that blood tests for the determination of DAO activity do not seem reasonable for the diagnosis of adverse reactions to histamine in food and beverages [13, 27]. 

## Is the differentiation between endogenous and exogenous histamine necessary? 

When the clinical picture and its diagnosis is discussed, there is frequently no differentiation between endogenously released and exogenously administered histamine. The fact that exogenous histamine below a toxic threshold is depleted and disposed of by intestinal DAO and perhaps also HNMT in healthy subjects as well as the lack of correlation between DAO in the blood and intestinal DAO suggest that the differentiation between an endogenous and an exogenous disturbance of depletion is reasonable or even necessary. A limited depletion capacity for endogenously released histamine (e.g., after an allergic reaction) is not necessarily associated with a limited catabolization of ingested histamine. Consequently, for diagnosis a clear and reproducible relation between ingestion of histamine-rich food and subsequent symptoms should be established [26]. 

## Diagnosis of adverse reactions to ingested histamine 

The key factor in the successful diagnosis of any adverse reactions is a thorough patient history. Beyond the documentation of the detailed information provided by the patient it is essential to systematically inquire and question the symptoms supposedly related to the adverse reaction and to establish an unambiguous relationship with the ingestion of histamine-rich food or beverages. The reproducibility of symptoms is of particular interest in this context. If the symptoms are not reproducible, it is advisable to identify possible concomitant factors (see below) that need to be present to trigger the symptoms and, on the other hand, to eliminate other causes (see Differential diagnosis). An important device for diagnostic work-up is a diet and symptom diary. The detailed daily documentation of consumed food and beverages including quantities and time specifications as well as the description of symptoms (also with time specifications) do not only complement the details provided in the patient history but can in some cases also invalidate them. 

## Differential diagnosis 

A proper differential diagnosis is essential for successful therapy. The diet and symptom diary are also very useful in this context. First and foremost symptoms of toxic nature should be excluded. A histamine content of 100 mg/kg fish is already considered critical. This threshold can easily be exceeded in spoiled fish belonging to a family with high histidine content (e.g., scombridae, clupeidae, engraulidae, coryphaenidae, scomberesocidae). Therefore, the European Union has established a limit of 100 mg/kg (maximum of 200 mg/kg) (Commission Regulation (EC)****No 2073/2005) [29]). 

Even more important for differential diagnosis are changes in the gastrointestinal tract. They can influence the metabolization of ingested histamine by an increased permeability as well as by an impaired function of the depleting enzymes. A relationship between intestinal permeability and chronic urticaria could already be demonstrated in the 1990s [11, 12]. Bühner et al. [4] added to this knowledge by carrying out a study on the use of a pseudoallergen-poor diet (which is also poor in biogenic amines) in patients with chronic urticaria: they could show that those patients benefited most from the diet whose permeability of the gastroduodenal and intestinal mucosa had been increased before. Improvement or complete disappearance of symptoms after the diet was associated with the normalization of the gastroduodenal permeability. Kuefner et al. [15] demonstrated that the activity of both enzymes (DAO and HNMT) was reduced in patients with food allergy as well as in patients with colon adenoma. Unfortunately, it was not studied whether the reduced enzyme activity influenced the tolerability of histamine-containing food. Experience with patients also shows that the gastroduodenal tract plays a major role in the development of adverse reactions to the ingestion of histamine-rich food. Patients with concomitant impairment of carbohydrate utilization or untreated gluten-sensitive enteropathy frequently report adverse reactions to histamine that vanish or become rarer after successful therapy of the underlying disease. It is suggested that also the composition of intestinal bacteria, which is also influenced by the composition of ingested foods, plays an important role. All components of food are microbially fermented in varying degrees and thus influence the microbiotic composition in the small intestine as well as in the colon. This, on the other hand, can influence the pH value and the morphology of the gastrointestinal tract, and thus also its permeability [3]. 

When gastrointestinal symptoms are present it should, however, also be considered that symptoms can be triggered by endogenous histamine released, for example, by intestinal mast cells. 

To date, no useful objective parameters for the diagnosis of adverse reactions to histamine have been found. To our current knowledge the determination of DAO in the blood is not really significant [13, 27]. The measurement of intestinal DAO activity might be a more reliable parameter [26], but as it requires a biopsy it is also more complicated. 

The gold standard for the diagnosis of adverse reactions is a double-blind, placebo-controlled provocation test. Until now no sensible provocation regime has been developed and evaluated. Efforts to establish such a regime failed due to the fact that at a dose of 75 mg half of the controls without suspected adverse reactions to histamine developed symptoms [31]. Obviously this dose is so close to the toxic level that no differentiation between affected and unaffected persons is possible. 

As a consequence, diagnosis can currently only be based on clinical criteria and thus to a large extent on establishing a clear relationship between patient history and reproducible reactions. 

## Therapy approach 

If the diagnosis “adverse reactions to exogenous histamine” is reproducible and confirmed, therapy aims at limiting the symptoms to a minimum without reducing the patient’s quality of life. General histamine-poor diets, such as those found on the web and frequently recommended to patients, are usually inappropriate to achieve this therapeutic goal (see below). At most, they can be used as diagnostic tools in order to make sure that the symptoms are absent during this diet. However, it is important not to forget the possible placebo effect that might occur from the patient’s hope for improvement. Therefore, the improvement of symptoms during diet alone is not a proof for underlying adverse reactions to histamine. 

## General histamine-poor diets are not sensible 

These kinds of diets are not suitable as a long-term therapy, mainly because they limit the range of food and beverages too much. The histamine contents of food can vary significantly: freshly caught fish, for example, contains almost no histamine, while the histamine content of cured, pickled or not really fresh fish can be more than 2,000 mg/kg [22]. The histamine content of cheese, another frequently cited histamine-rich food, can also vary significantly. Even Emmental cheese, Swiss mountain cheese or blue cheese can contain less than 5 mg/kg of histamine [1, 22]. Similar is true for sausages, wine, pickled vegetables and the like [1, 6, 20]. But not even the detailed knowledge of the histamine content of a certain food allows to draw conclusions about its tolerability. While increased amounts of histamine in fish can easily cause toxic reactions, considerably higher amounts of histamine are tolerated when consumed in the form of cheese [5]. Thus, the senate commission of the Deutsche Forschungsgemeinschaft (German Research Foundation) states that the maximum amount of 200 mg histamine/kg indicated in the hygiene regulation on fish cannot be applied to cheese. As a possible explanation they indicate a slower release of biogenic amines from cheese in the gastrointestinal tract. 

This statement is supported by experience from nutrition therapy. The individual tolerability is obviously significantly influenced by the choice of food as well as by the composition and intervals of meals. Low-carbohydrate, protein- and fat-rich nutrition – as it is frequently applied in patients with impaired carbohydrate utilization – leads to a significant improvement of symptoms. The positive effect is probably based on an increased gastrointestinal passage time, a changed permeability and subsequently a longer period of action of the catabolizing enzymes, but also on the influence on the patient’s intestinal microflora. Possibly the too high amounts of carbohydrates in a bread- or starch-rich diet can also explain why many patients report not to tolerate bread very well. Patient guides often ascribe this effect to the yeast used in bread baking, but the detectable values for yeast are clearly below 10 mg/kg. Yeast extract, on the other hand, can contain significant amounts of histamine. 

As histamine depletion is delayed due to the presence of other biogenic amines in food, also food with a high content of other biogenic amines is “banned” in many generalized diet lists. However, also the amounts of other biogenic amines vary according to ripeness, storage, preparation, deterioration, and the like, so that a general ban of food with a high content of biogenic amines that is not individually adjusted for each patient, is always associated with restrictions. 

Furthermore, the frequently recommended total avoidance of so-called histamine liberators limits the patient’s choice of food significantly. Whether histamine liberators, that only contain small amounts of histamine themselves but are supposed to trigger a histamine release really exist, is controversially discussed [30]. 

A survey of patients with suspected adverse reactions to histamine showed that a number of supposedly relevant foods are indeed not tolerated by many, but by far not by all, affected persons, while others can consume them without any problems [10]. This observation shows how important individual therapeutic guidance is. 

## Individual therapy for a better quality of life 

Therapeutically, a three-step approach, as also recommended by the Food Allergy Working Group of Deutsche Gesellschaft für Allergie und klinische Immunologie (DGAKI), has proven effective [21]. The initial avoidance phase of 10 – 14 days is primarily dedicated to symptom reduction by limiting the supply of biogenic amines. Additionally, the general choice of food, the composition of meals and the intervals between them are important. If the patient is used to a carbohydrate-rich diet, the addition of proteins and fat and the reduction of carbohydrates has proven to be an effective approach to support symptom reduction. After 2 weeks the avoided foods should be reintroduced (testing). This expands the variety of foods that can be consumed by the patient while taking into account individual factors (stress, hormone status, drug use and so on). The last phase is an individually adjusted continuous diet that warrants a high quality of life and does not limit the patient’s choice of food too much. 

## Conclusion 

The subjective suspicion of adverse reactions to histamine is – similarly to other adverse/intolerance reactions – by far more frequent than their objective detectability. What makes diagnosis and therapy even more difficult is the fact that diagnosis can be made only based on clinical observations. For diagnostic work-up it is essential to establish an objective relationship between patients’ statements and reproducible reactions. Therapeutically, it is indispensable to advise the affected people individually in order to limit their nutritional restrictions to a minimum and, thus, to maintain their quality of life. 

**Table 1.** Diagnostic work-up when adverse reactions to histamine in food and beverages is suspected (modified from [26]).

Detailed medical/dietary history
Verify association/reproducibility between ingestion of food and symptoms using a dietary and symptom protocol
Exclude other causes (intake of toxic amounts, impairment of carbohydrate utilization, celiac disease, delayed depletion of endogenous histamine, increased endogenous release and so on)
DAO determination – if useful at all, should be only determined in the intestine
Provocation testing

## References

[1] Askar A Biogene Amine in Lebensmitteln und ihre Bedeutung. Ernahr-Umsch. 1982; 29: 143–148.

[2] Bachert C The role of histamine in allergic disease: re-appraisal of its inflammatory potential. Allergy. 2002; 57: 287–296. 11906358 10.1034/j.1398-9995.2002.1r3542.x

[3] Blaut M Loh G Aufbau und Funktion der intestinalen Mikrobiota des Menschen In: Bischoff SC. Probiotika, Präbiotika und Synbiotika. Stuttgart: Thieme; 2009 2-22.

[4] Buhner S Reese I Kuehl F Lochs H Zuberbier T Pseudoallergic reactions in chronic urticaria are associated with altered gastroduodenal permeability. Allergy. 2004; 59: 1118–1123. 15355472 10.1111/j.1398-9995.2004.00631.x

[5] Eisenbrand G Biogene Amine in Käse und Fisch. DFG Senatskommission zur Beurteilung der gesundheitlichen Unbedenklichkeit von Lebensmitteln (SKLM). 1998; 10.1007/s00103-004-0902-8 15378173

[6] Ekici K Coskun H Histamine contents in some commercial vegetable pickles. Pak J Nutr. 2004; 3: 197–198.

[7] Fülöp AK Földes A Buzás E Hegyi K Miklós IH Romics L Kleiber M Nagy A Falus A Kovács KJ Hyperleptinemia, visceral adiposity, and decreased glucose tolerance in mice with a targeted disruption of the histidine decarboxylase gene. Endocrinology. 2003; 144: 4306–4314. 12960041 10.1210/en.2003-0222

[8] Jarisch R Leserbrief Allergologie. 2009; 32: 41–42.

[9] Jutel M Akdis M Akdis CA Histamine, histamine receptors and their role in immune pathology. Clin Exp Allergy. 2009; 39: 1786–1800. 20085595 10.1111/j.1365-2222.2009.03374.x

[10] Kamp A Ernährungstherapie der Histaminintoleranz. EM (Pittsburgh Pa). 2009; 24: 78–81.

[11] Kanny G Moneret-Vautrin DA Schohn H Feldman L Mallie JP Gueant JL Abnormalities in histamine pharmacodynamics in chronic urticaria. Clin Exp Allergy. 1993; 23: 1015–1020. 10779295 10.1111/j.1365-2222.1993.tb00293.x

[12] Kanny G Grignon G Dauca M Guedenet JC Moneret-Vautrin DA Ultrastructural changes in the duodenal mucosa induced by ingested histamine in patients with chronic urticaria. Allergy. 1996; 51: 935–939. 9020424 10.1111/j.1398-9995.1996.tb04497.x

[13] Kofler H Aberer W Deibl M Hawranek T Klein G Reider N Fellner N Diamine oxidase (DAO) serum activity: not a useful marker for diagnosis of histamine intolerance. Allergologie. 2009; 32: 105–109.

[14] Komericki P Klein G Hawranek T Land R Reider N Strimitzer T Kranzelbinder B Aberer W Oral verabreichte Diaminoxidase (DAO) bei Patienten mit Verdacht auf Histamin-Intoleranz. Allergologie. 2008; 31:190

[15] Kuefner MA Schwelberger HG Hahn EG Raithel M Decreased histamine catabolism in the colonic mucosa of patients with colonic adenoma. Dig Dis Sci. 2008; 53: 436–442. 17562176 10.1007/s10620-007-9861-x

[16] Maintz L Bieber T Novak N Die verschiedenen Gesichter der Histaminintoleranz. Dtsch Arztebl. 2006; 51-52: A3477–A3483.

[17] Maintz L Schwarzer V Bieber T van der Ven K Novak N Effects of histamine and diamine oxidase activities on pregnancy: a critical review. Hum Reprod Update. 2008; 14: 485–495. 18499706 10.1093/humupd/dmn014

[18] Mayer I Missbichler A Wantke F Focke M Reichl H Winter M Jarisch R Optimierter Radioassay zur quantitativen Bestimmung der Aktivität von Diaminooxidase (DAO) in humanem Serum und Plasma. Allergologie. 2005; 28: 1–8.

[19] Pali-Schöll I Herzog R Wallmann J Szalai K Brunner R Lukschal A Karagiannis P Diesner SC Jensen-Jarolim E Antacids and dietary supplements with an influence on the gastric pH increase the risk for food sensitization. Clin Exp Allergy. 2010; 40: 1091–1098. 20214670 10.1111/j.1365-2222.2010.03468.x PMC2999750

[20] Pechanek U Pfannhauser W Woidich H Untersuchung über den Gehalt biogener Amine in vier Gruppen von Lebensmitteln des österreichischen Marktes. Z Lebensm Unters Forsch. 1983; 176: 335–340. 6613353 10.1007/BF01057722

[21] Reese I Ballmer-Weber B Beyer K Erdmann S Fuchs T Kleine-Tebbe J Lepp U Henzgen M Niggemann B Saloga J Schäfer C Werfel T Zuberbier T Worm M Vorgehen bei Verdacht auf Unverträglichkeit gegenüber oral aufgenommenem Histamin. Stellungnahme der AG NMA der DGAKI. Allergo J. In press.

[22] Sarkadi L Histamine in food In: Falus A Grosman N Darvas Z Histamine: biology and medical aspects. Budapest - Basel: Springer Med Publishing Karger; 2004 176-185.

[23] Sattler J Häfner D Klotter HJ Lorenz W Wagner PK Food-induced histaminosis as an epidemiological problem: plasma histamine elevation and haemodynamic alterations after oral histamine administration and blockade of diamine oxidase (DAO). Agents Actions. 1988; 23: 361–365. 3134804 10.1007/BF02142588

[24] Sattler J Lorenz W Kubo K Schmal A Sauer S Lüben L Food-induced histaminosis under diamine oxidase (DAO) blockade in pigs: further evidence of the key role of elevated plasma histamine levels as demonstrated by successful prophylaxis with antihistamines. Agents Actions. 1989; 27: 212–214. 2568741 10.1007/BF02222242

[25] Sattler J Lorenz W Intestinal diamine oxidases and enteral-induced histaminosis: studies on three prognostic variables in an epidemiological model. J Neural Transm Suppl. 1990; 32: 291–314. 2128501 10.1007/978-3-7091-9113-2_39

[26] Schwelberger HG Histamine intolerance: a metabolic disease? Inflamm Res. 2010; 59: S219–S221. 20012758 10.1007/s00011-009-0134-3

[27] Töndury B Wüthrich B Schmid-Grendelmeier P Seifert B Ballmer-Weber B Histaminintoleranz: Wie sinnvoll ist die Bestimmung der Diaminoxidase-Aktivität im Serum in der alltäglichen klinischen Praxis? Allergologie. 2008; 31: 350–356.

[28] Untersmayr E Bakos N Schöll I Kundi M Roth-Walter F Szalai K Riemer AB Ankersmit HJ Scheiner O Boltz-Nitulescu G Jensen-Jarolim E Anti-ulcer drugs promote IgE formation toward dietary antigens in adult patients. FASEB J. 2005; 19: 656–658. 15671152 10.1096/fj.04-3170fje

[29] Verordnung (EG) Nr. 2073/2005 der Kommission vom 15. November 2005 über mikrobiologische Kriterien für Lebensmittel..

[30] Vlieg-Boerstra BJ van der Heide S Oude Elberink JNG Kluin-Nelemans JC Dubois AEJ Mastocytosis and adverse reactions to biogenic amines and histamine-releasing foods: what is the evidence? Neth J Med. 2005; 63: 244–249. 16093574

[31] Wöhrl S Hemmer W Focke M Rappersberger K Jarisch R Histamine intolerance-like symptoms in healthy volunteers after oral provocation with liquid histamine. Allergy Asthma Proc. 2004; 25: 305–311. 15603203

